# Is childhood trauma associated with loneliness, mental health symptoms and social exclusion in adulthood? A UK Biobank Study

**DOI:** 10.1192/j.eurpsy.2023.1128

**Published:** 2023-07-19

**Authors:** C. M. Van Der Feltz-Cornelis, S. Allen, K. Atkin, S. Gilbody

**Affiliations:** 1 Health Sciences, University of York, York; 2 Psychology, Northumbria University, Newcastle, United Kingdom

## Abstract

**Introduction:**

Childhood trauma has been linked to adult psychosocial outcomes including social exclusion, loneliness, and psychological distress.

**Objectives:**

To explore the associations between childhood trauma and social exclusion in adulthood with consideration of loneliness and symptoms of anxiety and depression in the UKBiobank database.

**Methods:**

Hierarchical multiple regression analysis of 87,545 participants (mean age=55.68[7.78], 55.0% female, 97.4% white) enrolled in the UK Biobank. The main predictor variable was occurrences of traumatic childhood experiences. Current loneliness and symptoms of anxiety (GAD-7) and depression (PHQ-9) were included as secondary predictors. The outcome variables were ‘limited social participation’, ‘area deprivation’, ‘individual deprivation’ and (combined) ‘social exclusion’ .

**Results:**

We found small associations between childhood trauma and social exclusion, explaining between 1.5% and 5.0% of the variance. Associations remained significant when loneliness, anxiety, and depression were entered in the models. These findings support a relationship between early-life adversity and socioeconomic deprivation including heightened risks of homelessness, antisocial behaviour and lower social mobility in adulthood.

Loneliness was the strongest predictor of ‘limited social participation.’

Depression was the strongest predictor of ‘individual deprivation,’ ‘area deprivation’ and ‘social exclusion,’ closely followed by childhood trauma.

Anxiety symptoms protected against ‘individual deprivation’ and ‘social exclusion’ in adulthood. Given the composition of the ‘individual deprivation’ dimension (i.e. employment, education, income) this may tentatively suggest that low levels of anxiety could have a positive impact on individuals’ pursuit of education and employment, potentially in line with the theory that the Yerks and Dodson law (i.e., there is an inverted U-shaped relationship between arousal and cognitive performance; Yerkes & Dodson, 1908) may apply to anxiety symptoms. In other words, higher vigilance may help seeking a way out of childhood adversity and increase cognitive performance if anxiety is low level, thus possibly playing a role in resilience. This may particularly be the case given that average levels of anxiety were low in the current sample (only 5% had a clinically significant GAD-7 score of above 10).

**Conclusions:**

Trauma and neglect in childhood are associated with an increase of social exclusion in adulthood.

Loneliness and depression make this association stronger.

Anxiety symptoms may lead to better performance in education and employment and hence play a protective role against individual level social deprivation - higher vigilance and cognitive performance can occur in low level anxiety and may increase resilience in adulthood.

**Disclosure of Interest:**

None Declared

